
JOURNAL INFORMATION
==============================
NLM Title Abbreviation: South Med Rec
NLM Journal ID: 0027317

The Southern Medical Record

Publisher: Biomedical Journal Digitization Project

ARTICLE INFORMATION
==============================
PMCID: PMC9085135
PMID: 36022845
Article version: 1
Subjects: Editorials and Miscellaneous

Prof. Loisette’s Memory Discovery

Print publication date: 1887 Dec
Volume: 17
Issue: 12
First page: 527
Last page: 527
License: This work is free of known copyright restrictions. For more information, please see PMC Back Issue Digitization.
License URL: https://creativecommons.org/publicdomain/mark/1.0/

==============================

RECEIPTED.—1887—Drs. T. B. Robertson, C. M. Bold, J. C. Moody, to July; H. Mobley, to March; J. A. Daniel, W. Brewington, W. S. Bradford, G. W. Bowling, J. F. Burnett, J. F. Davis. 1888—J. B. Long, to October; P. M. Catching, to July; M. M. Evans, to July; J. H. Wysong, to July.

PROF. LOISETTE'S MEMORY DISCOVERY.—Prof. Loisette's new system of memory training; taught by correspondence at 237 Fifth Avenue, New York, seems to supply a general want. He has had two classes at Yale of 200 each, 250 at Meriden, 300 at Norwich, 100 Columbia law students, 400 at Wellesley College, and 400 at University of Pennsylvania, etc. Such patronage and the endorsement of such men as Mark Twain, Dr. Buckley, Prof. Wm. R. Harper, of Yale, etc., place the claim of Prof. Loisette upon the highest ground.

---

### SPECIAL NOTES.

**Warner's Pill Chalybeate Compound.**—This pill contains carb. proloxide of iron and nux vomica, and is an admirable tonic and alterative in anæmia, chlorosis, phthisis, scrofula, loss of appetite. The physician may see that he is obtaining exactly what he prescrides by ordering in bottles containing one hundred each. Caution.—Specify Warner & Co., and see to the special marks and autograph on wrapper. May order by mail. Discount for quantities. Wm. R. Warner & Co., Philadelphia.

**Cerebral Sedative Compound** is a valuable hypnotic prepared by Parke, Davis & Co. It contains potassium bromide, chloral hydrate, gelsemium and opium. There is another preparation in which henbane is substituted for the opium. Descriptive circulars giving formulæ may be had by addressing Parke, Davis & Co., Detroit, Michigan.
